# Longevity of the insecticidal effect of three pyrethroid formulations applied to outdoor vegetation on a laboratory-adapted colony of the Southeast Asian malaria vector *Anopheles dirus*

**DOI:** 10.1371/journal.pone.0231251

**Published:** 2020-04-14

**Authors:** Victor Chaumeau, Praphan Wisisakun, Sunisa Sawasdichai, Prasan Kankew, Gay Nay Htoo, Somsak Saithanmettajit, Sarang Aryalamloed, Naw Yu Lee, Gilles Delmas, François Nosten

**Affiliations:** 1 Shoklo Malaria Research Unit, Mahidol-Oxford Tropical Medicine Research Unit, Faculty of Tropical Medicine, Mahidol University, Mae Sot, Thailand; 2 Centre for Tropical Medicine and Global Health, Nuffield Department of Medicine, University of Oxford, Oxford, England, United Kingdom; University of Saskatchewan College of Agriculture and Bioresources, CANADA

## Abstract

Outdoor residual spraying is proposed for the control of exophilic mosquitoes. However, the residual effect of insecticide mists applied to outdoor resting habitats of mosquitoes is not well characterized. The objective of this study was to assess the longevity of the residual insecticidal effect of three pyrethroid formulations applied to outdoor vegetation against the Southeast Asian malaria vector *Anopheles dirus*. Lambda-cyhalothrin capsule suspension, deltamethrin emulsifiable concentrate and bifenthrin wettable powder were sprayed on dense bamboo bushes on the Thailand-Myanmar border during the dry season 2018. The duration and magnitude of the residual insecticidal effect were assessed weekly with a standard cone assay, using freshly collected insecticide-treated bamboo leaves and a laboratory-adapted colony of *Anopheles dirus sensu stricto* susceptible to pyrethroids. The experiment was repeated during the rainy season to assess the persistence of the lambda-cyhalothrin formulation after natural rains and artificial washings. During the dry season (cumulative rainfall = 28 mm in 111 days), mortality and knockdown (KD) rates were >80% for 60 days with bifenthrin and 90 days with lambda-cyhalothrin and deltamethrin. The 50% knockdown time (TKD50) was <15 min with lambda-cyhalothrin and deltamethrin, and <30 min with bifenthrin. During the rainy season (cumulative rainfall = 465 mm in 51 days), mortality and KD rates were >80% for 42 days and TKD50 was <15 min with lambda-cyhalothrin. Additional artificial washing of the testing material with 10L of tap water before performing the cone tests had no significant effect on the residual insecticidal effect of this formulation. Long-lasting residual insecticidal effect can be obtained when spraying pyrethroid insecticides on the outdoor resting habitats of malaria vectors.

## Introduction

The two broadly scalable interventions recommended by World Health Organization (WHO) for malaria vector-control are mass distribution of long-lasting insecticide-impregnated bed nets (LLINs) and, where appropriate, indoor residual spraying (IRS) [1]. LLINs protect against mosquitoes seeking for human blood, indoors and at a time when people are sleeping under a bed net. Indoor residual spraying is effective against mosquitoes resting indoors, before or after the blood meal. However, these stereotypical trophic behaviors apply only to a minority of the dominant malaria vectors worldwide [2, 3]. In some endemic areas, LLINs and IRS have only a marginal impact on malaria [4, 5].

To avoid severe desiccation and heat stress during daytime, mosquitoes seek for resting habitats that provide a fresh and humid microclimate [6]. Daytime resting habitats have been identified both indoors (*e*.*g*. roof, wall, ceilings of houses and animal barns) and outdoors (*e*.*g*. tree holes, rodent holes, dense bushes, wells) [7]. Most mosquito species rest exclusively outside in natural settings, and only a relatively few species rest inside man-made shelters [7, 8]. Therefore, outdoor residual spraying has been proposed for the control of exophilic mosquitoes, including relevant malaria vectors [9–11].

Several published studies reported the duration and magnitude of the residual insecticidal effect of insecticide mists applied to outdoor vegetation (Table 1). After the studies on dichlorodiphenyltrichloroethane (DDT) in the 1940s, the main insecticide classes used for public health purposes were tested including pyrethroids (*e*.*g*. permethrin, deltamethrin, bifenthrin and lambda-cyhalothrin), organophosphate (*e*.*g*. malathion), carbamate (*e*.*g*. bendiocarb) and insect growth inhibitors (*e*.*g*. pyriproxyfen) [10–12]. Residual insecticidal effect lasting for 1 to 98 days was reported but the results are difficult to interpret because the assays were not standardized, and insecticide susceptibility of the mosquitoes used in the assay was not reported. The standard 3-minute exposure cone assay recommended by WHO for the evaluation insecticides used for IRS and treatment of mosquito bed-nets was used only in one study [11]. Most studies used a 24-hour exposure time, leading to an overestimation of the insecticidal effect [13]. Only two studies were conducted with malaria vectors [9, 10].

**Table 1:** Summary of published studies to characterize the duration and magnitude of the residual insecticidal effect of insecticide mists applied to outdoors vegetation.

Date	Place	Biotope	Insecticide (Formulation)^a^	Handling concentration in g a.i. /L^a^	Target dose in g a.i. /m^2^^a^	Mosquito species^a^^,^^b^	Exposure time	Outcome	T90^c^ in days	T50^c^ in days	Ref
**1945 (Jul)**	Florida, USA	Salt marsh	DDT (EC)	50	0.93	*An*. *quadrimaculatus*	24 hrs	Mortality/KD rate at the end of exposure	30	30	[9]
**1979 (Jun)**	Ontario, Canada	Lawn	permethrin + PPB (EC)	5	0.2	*Aedes* spp.	6 hrs	Mortality/KD rate at the end of exposure	19	26	[14]
**1989 (NR)**	Dominican Republic	Screen cage	permethrin (EC)	NR	0.012	*An*. *albimanus*	24 hrs	Mortality/KD rate at the end of exposure	>56	>56	[10]
**2004 (May)**	Florida, USA	Screen cage	deltamethrin (SC)	0.55	NR	*Ae*. *albopictus*, *Cx*. *quinquefasciatus*	24 hrs	Mortality/KD rate at the end of exposure	>84	>84	[15]
**2004 (Jun)**	Kentucky, USA	Suburban backyards	λ-cyhalothrin (CS)	0.6	0.016	*Ae*. *albopictus*	24 hrs	Mortality/KD rate at the end of exposure	7	28	[16]
**2005 (May)**	Florida, USA	Screen cage	λ-cyhalothrin (CS)	0.3	NR	*Ae*. *albopictus*, *Cx*. *quinquefasciatus*	24 hrs	Mortality/KD rate at the end of exposure	>84	>84	[17]
**2006 (Aug)**	Florida, USA	Screen cage	bifenthrin (WP)	0.6	0.025	*Ae*. *albopictus*	1 hr	Mortality/KD rate at the end of exposure	0	7	[13]
**2006 (Sep)**	Florida, USA	Public park	bifenthrin (WP)	0.6	0.025	*Ae*. *albopictus*, *Cx*. *quinquefasciatus*	24 hrs	Mortality/KD rate at the end of exposure	21	28	[18]
**2007 (Jul)**	Florida, USA	Suburban backyards	permethrin (EC)	NR	0.016	*Cx*. *quinquefasciatus*	24 hrs	Mortality/KD rate at the end of exposure	7	14	[19]
**2008 (Mar)**	California, USA	Desert	bifenthrin (WP)	0.6	0.025	*Cx*. *tarsalis*	24 hrs	Mortality/KD rate at the end of exposure	1	14	[20]
**2012 (NR)**	Queensland, Australia	Suburban backyards	bifenthrin (WP)	1	0.1	*Ae*. *vigilax*	30 min	Mortality 24 hrs after exposure	>56	>56	[21]
**2012 (Mar)**	Queensland, Australia	Forest	λ-cyhalothrin (CS)	0.4	NR	*Ae*. *aegypti*	24 hrs	Mortality/KD rate at the end of exposure	>98	>98	[22]
**2013 (NR)**	Catalonia, Spain	Screen cage	deltamethrin (EC)	0.2	0.010	*Ae*. *albopictus*	24 hrs	Mortality/KD rate at the end of exposure	12	27	[23]
**2013 (Oct)**	Florida, USA	Swamp	bifenthrin (WP)	0.6	0.025	*Ae*. *aegypti*	24 hrs	Mortality/KD rate at the end of exposure	7	>28	[24]
**2017 (Aug)**	New Jersey, USA	Suburban backyards	λ-cyhalothrin (CS)	0.6	NR	*Ae*. *albopictus*	3 min	Mortality 24 h after exposure	7	28	[11]

*Abbreviations*: CS, capsule suspension; DDT, dichlorodiphenyltrichloroethane; EC, emulsion concentrate; KD, knockdown; NR, not reported; PPB, piperonyl butoxide; Ref, references; SC, suspension concentrate; WP, wettable powder.

^a^If several experimental conditions were compared in a given study, only the condition leading to the most successful outcome was reported in the table.

^b^Mosquito species used to perform the assay. Laboratory-adapted mosquito colonies were used in all studies except in reference [14] for which tests were performed with wild caught female imagoes.

^c^T90 and T50 are the time necessary for the mortality rate to drop below 90% and 50% respectively.

The objective of this study was to assess the longevity of the residual insecticidal effect of three different pyrethroid formulations applied to outdoor vegetation, using a standard cone assay and a pyrethroid-susceptible laboratory-adapted colony of *Anopheles dirus*, a highly exophilic dominant malaria vector in mainland Southeast Asia.

## Methods

### Study design

The study was conducted in enclosed experimental areas on the Thailand-Myanmar border. A first experiment was conducted in January 2018 to assess the longevity of the insecticidal effect of three formulations of pyrethroid insecticides applied to outdoor vegetation during the dry season. The insecticides were the 2.5% capsule suspension (CS) of lambda-cyhalothrin Karate Zeon^®^ 2.5 CS (Syngenta, Basel, Switzerland), the 2% concentrated aqueous emulsion (EW) of deltamethrin Aqua K-Othrine^®^ EW (Bayer, Leverkusen, Germany) and the 10% wettable powder (WP) of bifenthrin Bistar^®^ 10 WP (FMC, Philadelphia, USA). Each insecticide was diluted in tap water at a final handling concentration of 2 g a.i. /L, and sprayed on bamboo bushes with a mist blower model PM7650H (Makita, Anjo, Japan) at a target concentration of 500 g a.i. /ha. A standard cone assay was used to assess the residual insecticidal effect of insecticide-treated bamboo leaves against a colony of pyrethroid-susceptible laboratory-adapted *An*. *dirus* [25]. Tests were carried out weekly until mortality and KD rates dropped below 80%.

A second experiment was conducted in May 2018 (beginning of the rainy season) to assess the effect of natural rains and artificial washes on the longevity of the insecticidal effect of the lambda-cyhalothrin formulation. The insecticide was handled at a concentration of 2 g a.i. /L and sprayed on bamboo bushes with a mist blower model PM7650H at a target concentration of 500 g a.i. /ha. Residual insecticidal effect was assessed weekly until mortality and KD rates dropped below 80%. In this experiment, half of the bamboo leaves were washed thoroughly with 10 L of tap water and allowed to dry for 3 hours before being tested with the cone assay. Freshly collected leaves were hung on a clothes horse and hosed down with a watering can for 30 seconds. The other half was tested without being washed. Experimental areas of the two experiments were spaced 5 kilometers one from another, and consisted in plots of 500 m^2^ covered with bamboo bushes separated from each other by 50 meters of wasteland (one plot per experimental condition). The species of bamboo used in the experiments was *Gigantochloa ablociliata*. Bamboo was chosen because it is often associated with *Anopheles dirus* populations [26]. Leaves were selected at random for all experiments. Meteorological data were obtained from the Thai Meteorological Department.

### *Anopheles dirus* colony

The stenogamous colony of *Anopheles dirus sensu stricto* used in this study originated from Cambodia. It was established decades ago and has never been selected for insecticide resistance since. The colony identification was confirmed by Sanger sequencing of the cytochrome oxidase I gene (S1 Text and S1 Table; GenBank accession number: MT246865). Rearing conditions were as follow: imagoes were kept into 30 cm × 30 cm × 30 cm cages at a density of 1,000 specimen /cage and fed with a cotton pad soaked with a solution of 10% refined sugar and 0.5% of Multivitamin Syrup^®^ (Seven Seas, Bangkok, Thailand). Each cage was covered with a wet towel overlaid with a black plastic sheet. The insectarium was maintained at 27 ± 2 °C and 70–80% relative humidity, and illumination from fluorescent lighting was provided for 12 hours a day. Five to seven days after the emergence, female imagoes were fed on heparinized human blood. Gravid females were allowed to lay eggs in an ovoposition cup (7cm diameter × 4 cm depth) lined with wet filter paper and placed in the cage for 2 consecutive nights 3 days after the blood meal. Eggs were transferred every morning into a white plastic tray (25 cm × 36 cm × 6 cm) filled with 1.5L of drinkable water. Larvae were adjusted at a density not exceeding 100 specimen /tray and fed twice a day with finely grinded TetraBits Complete^®^ fish food (Tetra, Blacksburg, USA). Water was changed every three days until pupation (8–10 days after the eggs hatched). Pupae were collected in a plastic cup (7 cm diameter × 4 cm depth) filled with 100 mL of drinkable water at a density of 250 pupae per cup, and put in an empty cage for two days until emergence.

### Susceptibility assay

A standard susceptibility assay was used to determine the susceptibility of the mosquito colony to pyrethroid insecticides [27]. Female imagoes aged 3–5 day-old were exposed for 60 minutes to filter papers impregnated with 18 mg of deltamethrin /m^2^, 275 mg of permethrin /m^2^ or 18 mg of lambda-cyhalothrin /m^2^ into standard plastic cylinders (25 mosquitoes per tube). The number of mosquitoes knocked down was recorded every 5 minutes for 60 minutes. Mosquitoes were then transferred into standard holding tubes and provided with a 10% sugar solution. Mortality was recorded 24 hours after exposure to the insecticide. Mosquitoes exposed for 1 hour to a paper impregnated with the carrier (Dow 556 mixed with acetone) were used as a control. There were four insecticide-exposed replicates and two control replicates for each experimental condition.

### Cone assay

Freshly collected insecticide-treated bamboo leaves were laid on a panel of acrylic tilted at 45° and covered with ten standard plastic cones (10 replicates). Five female imagoes aged 3–5 day-old were introduced into each cone. After 3 minutes of exposure to the testing material, mosquitoes were transferred into 150-mL plastic cups (5 mosquitoes per cup) and provided with a 10% sugar solution. The number of mosquitoes knocked down was recorded every 5 minutes for 60 minutes. Mortality was recorded 24 hours after insecticide exposure. Bamboo leaves collected in a bush 50 meters outside of the area treated with the insecticide were used as a control for each experiment. Tests were conducted to verify that the mortality in the treated bushes was nil the day before the intervention. Cone assays and susceptibility assays were performed at 25 ± 2 °C with a relative humidity of 70–80%. All insecticide testing materials used in this study were provided by the Vector Control Research Unit (VCRU), Universiti Sains Malaysia.

### Data analysis

Knockdown (KD) rate was defined as the number of mosquitoes knocked down divided by the number of exposed mosquitoes. Knockdown time 50% (KDT50) was defined as the time after which 50% of the mosquitoes were knocked down. Mortality rate was defined as the number of mosquitoes dead at the end of the 24-hour observation time divided by the number of exposed mosquitoes. KDT50s and corresponding 95% confidence intervals (CIs) were estimated by fitting n-parameters logistic models to the KD kinetics with the *nplr* package version 0.1.7 of the R software [28] Exact binomial CIs were estimated for proportions (KD and mortality rates) with *epitools* package version 0.5–10 of the R software [29]. The mortality in the control was used to adjust the mortality in the insecticide treated group with Abbott’s formula [30].

## Results

### Susceptibility phenotype of the *Anopheles dirus* colony to deltamethrin, permethrin and lambda-cyhalothrin

**Fig 1:**
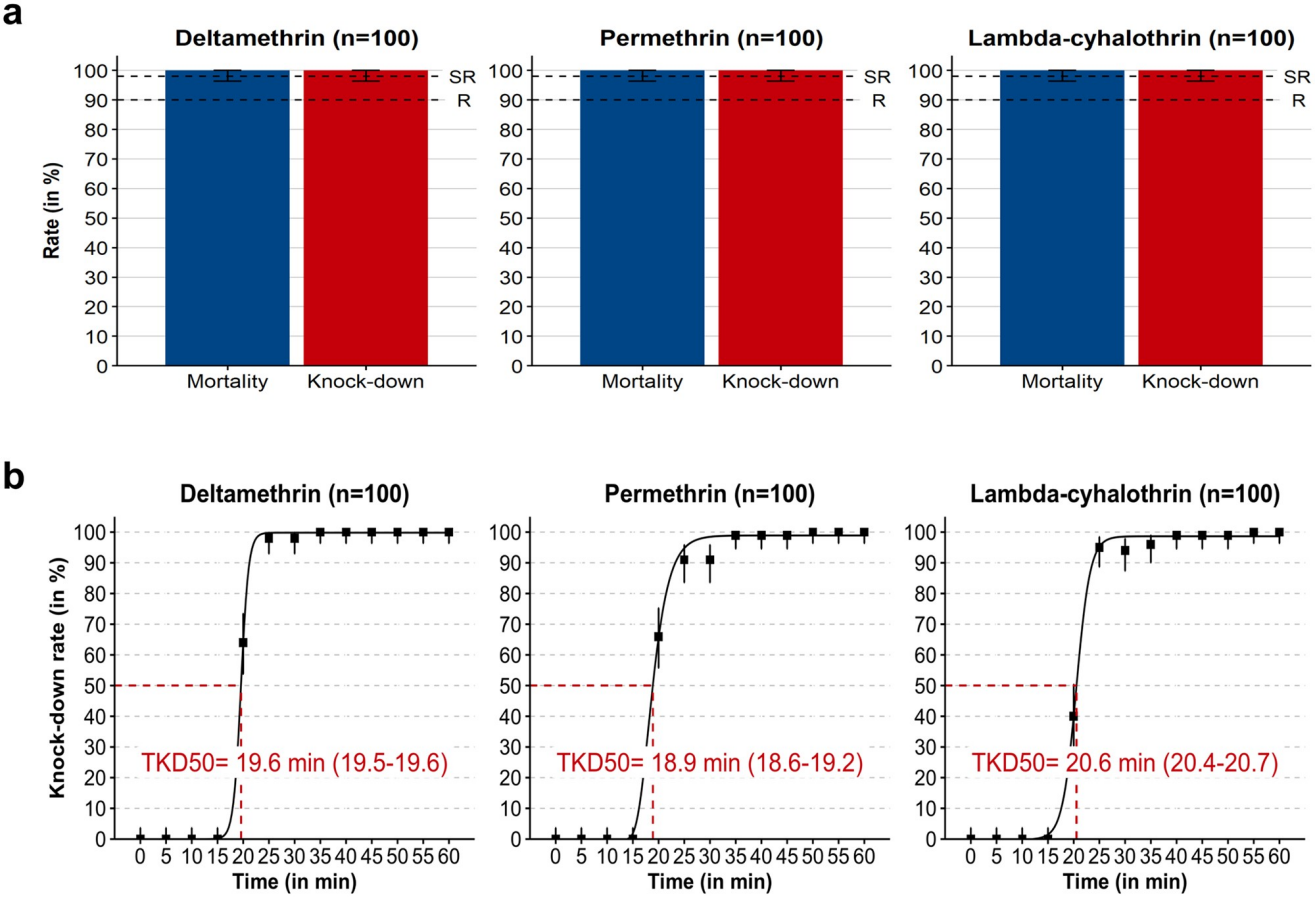
Phenotypic response of the *An*. *dirus sensu stricto* colony to pyrethroid exposure determined with a standard susceptibility assay. (a) KD rate at the end of the 60-minute exposure to insecticides and the mortality rate determined 24 hours after exposure to insecticides. (b) KD kinetic determined during the 1-hour insecticide exposure time. Mosquitoes were exposed to filter papers impregnated with 18 mg of deltamethrin /m^2^, 275 mg of permethrin /m^2^ or 18 mg of lambda-cyhalothrin /m^2^. The mosquito strain was considered susceptible to the insecticide tested if the mortality was ≥ 98% and resistant if the mortality was <90%. Suspected resistance was defined by an intermediate mortality rate. KDT50s and corresponding 95% CIs were estimated by fitting n-parameters logistic models to KD kinetics. Error bars indicate exact binomial 95% CIs. *Abbreviations*: CI, confidence interval; KD, knockdown; KDT50, 50% knockdown time; R, confirmed resistance; SR, suspected resistance.

The *An*. *dirus* colony used in this study was susceptible to deltamethrin, permethrin and lambda-cyhalothrin (Fig 1). KD and mortality rates in the susceptibility assay were 100% with the three insecticides. The KDT50 was 18.9, 19.6 and 20.6 min with permethrin, deltamethrin and lambda-cyhalothrin respectively. There was no mortality in the control batches.

### Longevity of the residual insecticidal effect of three pyrethroid formulations during the dry season

**Fig 2:**
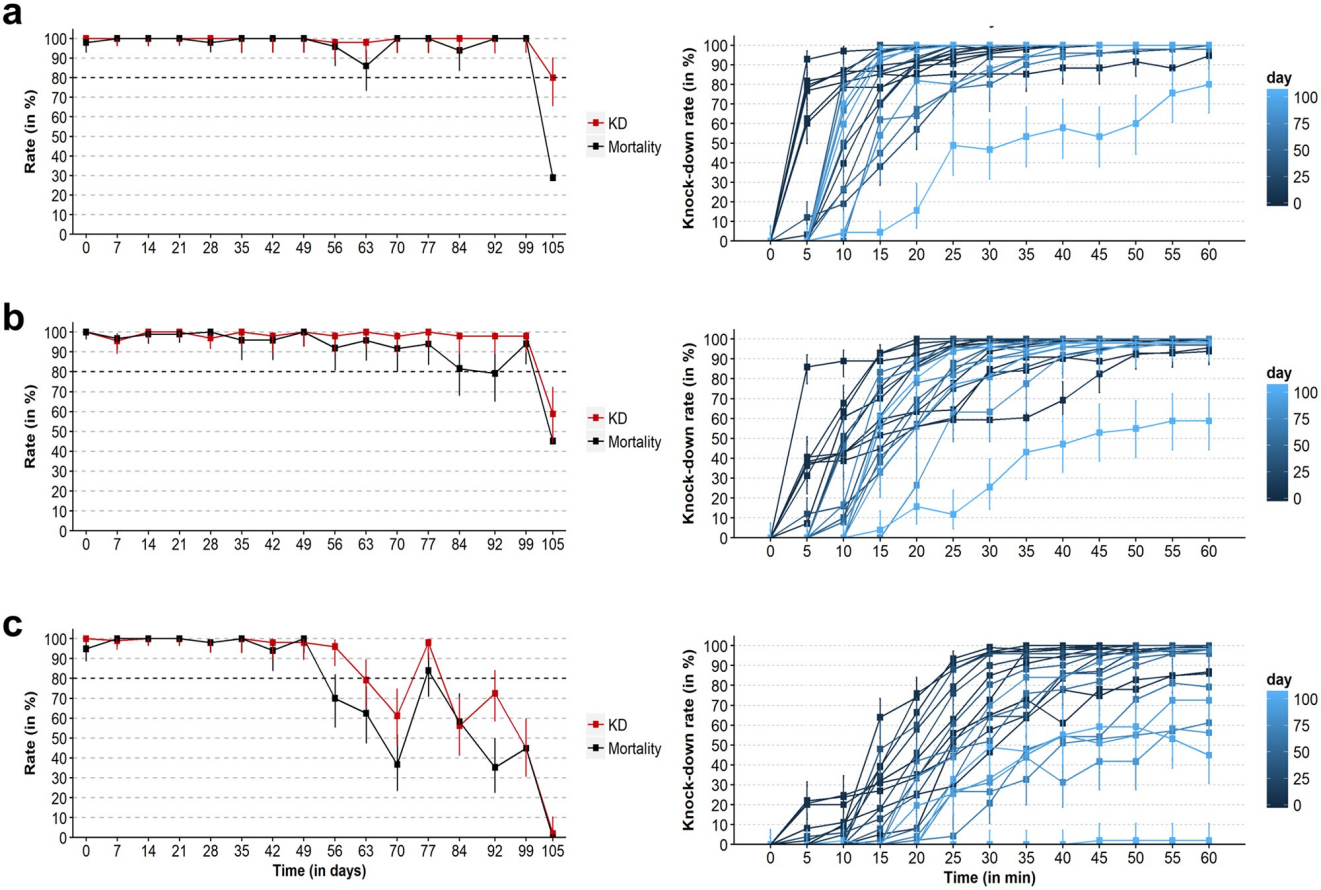
Duration and magnitude of the insecticidal effect of the three pyrethroid formulations applied to outdoor vegetation during the dry season. (A) Lambda-cyhalothrin CS. (B) Deltamethrin EW. (C) Bifenthrin WP. All insecticides were handled at a concentration of 2 g/L and sprayed a target concentration of 500 g of a.i. /ha on bamboo bushes in January 2018. Residual insecticidal effect was assessed with a standard cone assay using freshly collected insecticide-bamboo leaves and a laboratory-adapted colony of *An*. *dirus sensu stricto* susceptible to pyrethroid insecticides. Mortality and KD rates are shown on the left panels. KD kinetic is shown on the right panels. Error bars indicate exact binomial 95% CIs. *Abbreviations*: CS, capsule suspension; EW, concentrated aqueous emulsion; KD, knockdown, WP, wettable powder.

**Fig 3:**
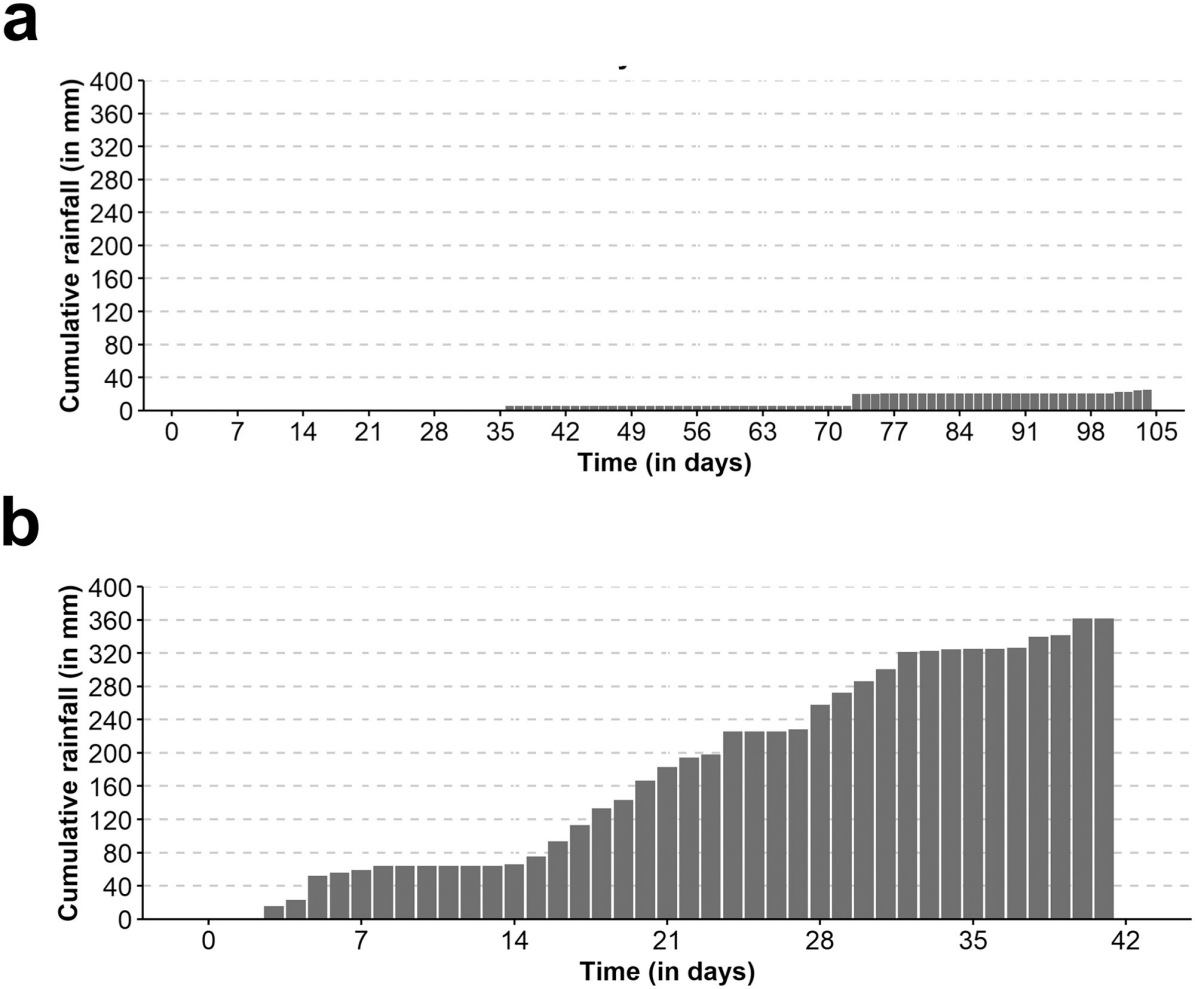
Cumulative rainfall recorded during the study. (A) Dry season. (B) Rainy season.

Long-lasting residual effects were obtained with all insecticide formulations during the dry season. The mortality and KD rates of lambda-cyhalothrin and deltamethrin were >80% for 98 days. The duration of the residual effect was shorter with bifenthrin, although mortality and KD rates were >80% for 49 days. Lambda-cyhalothrin gave the shortest KTD50s followed by deltamethrin and bifenthrin: 13/16, 9/16 and 1/16 data points with KDT50 <15 min respectively (Fig 2). Mortality in the controls was always less than 6%. The mean temperature was 27°C (range = 13–40°C) and the daily amplitude was 15°C in average. The mean relative humidity was 62% (range = 18–98%) and the daily amplitude was 47% in average. There were 9/111 rainy days during the follow-up and the cumulative rainfall was 28 mm (range of positive daily rainfall = 0.1–15 mm) (Fig 3).

### Effects of natural rains and artificial washes on the persistence of the lambda-cyhalothrin formulation

**Fig 4:**
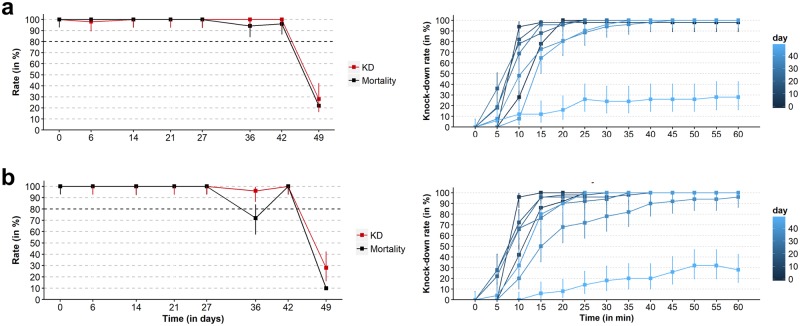
Duration and magnitude of the insecticidal effect of the lambda-cyhalothrin CS formulation applied to outdoor vegetation during the rainy season without additional artificial washing of the testing material (a) and with washing with 10 L of tap water before performing the cone assays (b). Insecticide was handled at a concentration of 2 g a.i. /L and sprayed a target concentration of 500 g of a.i. /ha on bamboo bushes in May 2018 (beginning of the rainy season). Residual insecticidal effect was assessed with a standard cone assay using freshly collected insecticide-bamboo leaves and a laboratory-adapted colony of *An*. *dirus sensu stricto* susceptible to pyrethroid insecticides. Mortality and KD rates are shown on the left panels. KD kinetic is shown on the right panels. Error bars indicate exact binomial 95% CIs. *Abbreviations*: CS, capsule suspension; KD, knockdown.

During the rainy season, the mortality and KD rates with lambda-cyhalothrin were >80% for 42 days, and the KDT50 ranged between 5 and 15 minutes. Similar results were obtained when the testing material was washed with 10L of tap water before performing the cone assay, although mortality dropped to 72% at day 36 (Fig 4). Mortality in the controls was always less than 4%. The mean temperature was 26°C (range = 22–38°C) and the daily amplitude was 8°C in average. The mean relative humidity was 83% (range = 43–98%) and the daily amplitude was 27% in average. There was 41/51 rainy days during the follow-up and the cumulative rainfall was 463 mm (range of positive daily rainfall = 0.1–34 mm) (Fig 3). Unlike in the dry season experiment, KD and mortality and rates declined sharply from 100% at day 42 to 10% at day 49 without apparent association with daily rainfalls.

## Discussion

We assessed the duration and magnitude of the residual insecticidal effect of three different pyrethroid formulations sprayed at a target dose of 500 g a.i. /ha on outdoor vegetation during the dry season, and additional data were presented for one of the formulation during the rainy season. High mortality and rapid KD were observed with a colony of pyrethroid-susceptible laboratory-adapted *An*. *dirus sensu stricto* exposed for 3 minutes to insecticide-treated bamboo leaves, up to 14 weeks after the spraying during the dry season and to 6 weeks during the rainy season. This study confirms that residual insecticidal effect compatible with malaria vector-control operation can be obtained when applying an insecticide mist to outdoor vegetation, and that a lethal concentration of the active ingredient can persist for several weeks despite monsoon rains with the lambda-cyhalothrin formulation.

Our results were similar to that reported during the evaluation of LLINs impregnated with 55 mg of deltamethrin /m^2^ (KD rate at 60 min = 100%, mortality = 100% and KDT50 < 10 min) [31]. It was not possible to do a simple comparison between our results and that of the published literature because the assays used to assess the residual insecticidal effect were not standardized until recently. However, the insecticidal effects lasted longer in this study than those reported in the Table 1, given that longer exposures to insecticide-treated materials used in previous studies overestimated assay outputs [13]. This difference is probably explained by the methodology used to spray insecticide mists (equipment, and handling and target concentrations of the active ingredients). Interestingly, the drop of KD and mortality rates was sharper during the rainy season than during the dry season experiment, and was not explained by rainfalls. This observation suggests that other factors may play a crucial role in the decay of insecticide concentration on plant surfaces, although the challenge in applying the insecticide mist homogeneously should also be considered.

The residual effect of lambda-cyhalothrin CS and deltamethrin EW formulations lasted longer and was stronger than that of the bifenthrin WP formulation. This difference may be explained by a shorter persistence of the WP formulation on treated surfaces when compared to more engineered CS and EW formulations, or by a lower intrinsic activity of bifenthrin when compared to deltamethrin and lambda-cyhalothrin [32]. Moreover, cone assay outputs reflect complex interactions between the active ingredient and the mosquitoes including KD, mortality and irritancy. Hougard *et al*. described in details the effects of seven pyrethroids on mosquitoes under laboratory conditions [33]. Exposing susceptible *An*. *gambiae* to operational doses of lambda-cyhalothrin, deltamethrin and bifenthrin (20–25 mg/m^2^) in the standard cone assay resulted in a strong and rapid KD effect (KD rate at 60 min = 100% and KDT50 between 8–12 min). Although fully susceptible mosquitoes were exposed, mortality rate ranged from 42% with lambda-cyhalothrin to 100% with deltamethrin. This result was explained by the interaction between irritant and lethal effects (irritancy preventing the contact between mosquitoes and the insecticide and therefore decreasing the mortality rate). Bifenthrin has lower irritant properties than other pyrethroids, which may favor the lethal effect rather than deterrence during operational deployments of vector-control interventions. In contrast, lambda-cyhalothrin is irritant to both pyrethroid susceptible and resistant *Anopheles* mosquitoes.

There were some limitations to the study. We did not assess the persistence of the deltamethrin and bifenthrin formulations during the rainy season and the residual insecticidal effect of insecticide mists on pyrethroid-resistant mosquitoes. Resistance to deltamethrin and permethrin in malaria vector populations collected in the Thailand-Myanmar border area has been reported [34] and the impact of pyrethroid resistances on the outcome of outdoor residual spraying remains to be determined. In addition, we did not assess the toxicity of insecticide mists and their residues against the environment and non-target organisms. Future research may aim at testing different insecticides, doses, materials, organisms (including pyrethroid resistant and wild malaria mosquitoes, as well as non-target organisms) and environmental conditions. Moreover, a better understanding of the rate and mechanisms of insecticide degradation on plant surfaces would provide a rational for optimizing insecticide formulation and increase the longevity of the residual effect. Finally, operational deployment of outdoor residual spraying will need further assessment of its impact on the entomological indices, diseases epidemiology and toxicity to environment.

## Conclusions

Long-lasting residual insecticidal effect can be achieved when spraying pyrethroid insecticides on outdoor resting habitats of anopheline mosquitoes. Outdoor residual spraying may therefore be used for the control of exophilic malaria vectors.

## Supporting information

S1 Text COI sequence of the *Anopheles dirus sensu* stricto colony determined by Sanger sequencing. (DOCX)

S1 Table Result of the COI sequence analysis with the Blast algorithm ran against the National Center for Biotechnology Information library. (DOCX)

S2 Table Data set of the susceptibility tests. (XLSX)

S3 Table Data set of the cone tests carried out during the dry season. (XLSX)

S4 Table Data set of the cone tests carried out to during the rainy season. (XLSX)

S5 Table Data set of the meteorological records. (XLSX)

## References

[1] World Health Organization. Core vector control methods 2015 [https://www.who.int/malaria/areas/vector_control/core_methods/en/].

[2] Kiszewski A, Mellinger A, Spielman A, Malaney P, Sachs SE, Sachs J. A global index representing the stability of malaria transmission. Am J Trop Med Hyg. 2004;70(5):486–98. 15155980

[3] Killeen GF. Characterizing, controlling and eliminating residual malaria transmission. Malar J. 2014;13:330 10.1186/1475-2875-13-330 25149656 PMC4159526

[4] Durnez L, Coosemans M. Residual malaria transmission: an old issue for new approaches In: Maguin S, editor. *Anopheles* mosquitoes—New insights into malaria vectors. Rijeka: IntechOpen; 2013.

[5] Chaumeau V, Fustec B, Nay Hsel S, Montazeau C, Naw Nyo S, Metaane S, et al. Entomological determinants of malaria transmission in Kayin state, Eastern Myanmar: A 24-month longitudinal study in four villages. Wellcome Open Res. 2018;3:109 10.12688/wellcomeopenres.14761.4 31206035 PMC6544137

[6] Dewald JR, Fuller DO, Muller GC, Beier JC. A novel method for mapping village-scale outdoor resting microhabitats of the primary African malaria vector, *Anopheles gambiae*. Malar J. 2016;15(1):489 10.1186/s12936-016-1534-9 27659918 PMC5034649

[7] Silver JB. Mosquito Ecology—Field Sampling Methods: Springer Netherlands; 2008.

[8] Sinka ME, Bangs MJ, Manguin S, Chareonviriyaphap T, Patil AP, Temperley WH, et al. The dominant *Anopheles* vectors of human malaria in the Asia-Pacific region: occurrence data, distribution maps and bionomic precis. Parasit Vectors. 2011;4:89 10.1186/1756-3305-4-89 21612587 PMC3127851

[9] Madden AH, Shoeder HO, Lindquist AW. Residual sprays applications to salt-marsh and jungle vegetation for control of mosquitoes. Journal of economic entomology. 1947;40(1):119–23. 10.1093/jee/40.1.119 20240407

[10] Perich MJ, Tidwell MA, Dobson SE, Sardelis MR, Zaglul A, Williams DC. Barrier spraying to control the malaria vector *Anopheles albimanus*: laboratory and field evaluation in the Dominican Republic. Med Vet Entomol. 1993;7(4):363–8. 10.1111/j.1365-2915.1993.tb00706.x 8268492

[11] Unlu I, Williams GM, Rochlin I, Suman D, Wang Y, Chandel K, et al Evaluation of Lambda-Cyhalothrin and Pyriproxyfen Barrier Treatments for *Aedes albopictus* (Diptera: Culicidae) Management in Urbanized Areas of New Jersey. J Med Entomol. 2018;55(2):472–6. 10.1093/jme/tjx216 29244157

[12] Richards SL, Volkan JK, Balanay JAG, Vandock K. Evaluation of Bifenthrin and Deltamethrin Barrier Sprays for Mosquito Control in Eastern North Carolina. J Med Entomol. 2017;54(6):1659–65. 10.1093/jme/tjx152 28968745

[13] Doyle MA, Kline DL, Allan SA, Kaufman PE. Efficacy of residual bifenthrin applied to landscape vegetation against *Aedes albopictus*. J Am Mosq Control Assoc. 2009;25(2):179–83. 10.2987/08-5804.1 19653500

[14] Helson B, Surgeoner G. Permethrin as a residual lawn spray for adult mosquito control. Mosquito News. 1983;43:164–9.

[15] Cilek JE, Hallmon CF. Residual effectiveness of pyrethroid-treated foliage against adult *Aedes albopictus* and *Culex quinquefasciatus* in screened field cages. J Am Mosq Control Assoc. 2006;22(4):725–31. 10.2987/8756-971X(2006)22[725:REOPFA]2.0.CO;2 17304943

[16] Trout RT, Brown GC, Potter MF, Hubbard JL. Efficacy of two pyrethroid insecticides applied as barrier treatments for managing mosquito (Diptera: Culicidae) populations in suburban residential properties. J Med Entomol. 2007;44(3):470–7. 10.1603/0022-2585(2007)44[470:eotpia]2.0.co;2 17547233

[17] Cilek JE, Hallmon CF. Residual effectiveness of three pyrethroids on vegetation against adult *Aedes albopictus* and *Culex quinquefasciatus* in screened field cages. J Am Mosq Control Assoc. 2008;24(2):263–9. 10.2987/5653.1 18666535

[18] Cilek JE. Application of insecticides to vegetation as barriers against host-seeking mosquitoes. J Am Mosq Control Assoc. 2008;24(1):172–6. 18437835 10.2987/8756-971X(2008)24[172:AOITVA]2.0.CO;2

[19] Amoo AO, Xue RD, Qualls WA, Quinn BP, Bernier UR. Residual efficacy of field-applied permethrin, d-phenothrin, and resmethrin on plant foliage against adult mosquitoes. J Am Mosq Control Assoc. 2008;24(4):543–9. 10.2987/08-5783.1 19181063

[20] Britch SC, Linthicum KJ, Wynn WW, Walker TW, Farooq M, Smith VL, et al. Evaluation of barrier treatments on native vegetation in a southern California desert habitat. J Am Mosq Control Assoc. 2009;25(2):184–93. 10.2987/08-5830.1 19653501

[21] Hurst TP, Ryan PA, Kay BH. Efficacy of residual insecticide Biflex AquaMax applied as barrier treatments for managing mosquito populations in suburban residential properties in southeast Queensland. J Med Entomol. 2012;49(5):1021–6. 10.1603/me11278 23025182

[22] Muzari OM, Adamczyk R, Davis J, Ritchie S, Devine G. Residual effectiveness of lambda-cyhalothrin harbourage sprays against foliage-resting mosquitoes in north Queensland. J Med Entomol. 2014;51(2):444–9. 10.1603/me13141 24724295

[23] Bengoa M, Eritja R, Lucientes J. Laboratory tests of the residual effect of deltamethrin on vegetation against *Aedes albopictus*. J Am Mosq Control Assoc. 2013;29(3):284–8. 10.2987/13-6331R.1 24199504

[24] Fulcher A, Farooq M, Smith ML, Li CX, Scott JM, Thomson E, et al. Evaluation of a new spraying machine for barrier treatment and penetration of bifenthrin on vegetation against mosquitoes. J Am Mosq Control Assoc. 2015;31(1):85–92. 10.2987/14-6424R.1 25843180

[25] World Health Organization. Guidelines for laboratory and field testing of long-lasting insecticidal mosquito nets. Geneva: World Health Organization; 2005.

[26] Suwonkerd W, Ritthison W, Ngo CT, Tainchum K, Bangs MJ, Chareonviriyaphap T. Vector biology and malaria transmission in Southeast Asia In: Manguin S, editor. *Anopheles* mosquitoes—New insights into malaria vectors. Rijeka: IntechOpen; 2013.

[27] World Health Organization. Test procedures for insecticide resistance monitoring in malaria vector mosquitoes Geneva: World Health Organization; 2016.

[28] Commo F, Bot BM. nplr: N-Parameter Logistic Regression. R package version 0.1–7. 2016.

[29] Aragon TJ. epitools: Epidemiology Tools. R package version 0.5–10. 2017.

[30] Abbott WS. A method of computing the effectiveness of an insecticide. 1925. J Am Mosq Control Assoc. 1987;3(2):302–3. 3333059

[31] Strode C, Donegan S, Garner P, Enayati AA, Hemingway J. The impact of pyrethroid resistance on the efficacy of insecticide-treated bed nets against African anopheline mosquitoes: systematic review and meta-analysis. PLoS Med. 2014;11(3):e1001619 10.1371/journal.pmed.1001619 24642791 PMC3958359

[32] Hougard JM, Zaim SD, Guillet P. Bifenthrin: a useful pyrethroid insecticide for treatment of mosquito nets. J Med Entomol. 2002;39(3):526–33. 10.1603/0022-2585-39.3.526 12061451

[33] Hougard JM, Duchon S, Darriet F, Zaim M, Rogier C, Guillet P. Comparative performances, under laboratory conditions, of seven pyrethroid insecticides used for impregnation of mosquito nets. Bull World Health Organ. 2003;81(5):324–33. 12856050 PMC2572461

[34] Chaumeau V, Cerqueira D, Zadrozny J, Kittiphanakun P, Andolina C, Chareonviriyaphap T, et al. Insecticide resistance in malaria vectors along the Thailand-Myanmar border. Parasit Vectors. 2017;10(1):165 10.1186/s13071-017-2102-z 28359289 PMC5374572

