# Supplementary material for: Longevity of the insecticidal effect of three pyrethroid formulations applied to outdoor vegetation on a laboratory-adapted colony of the Southeast Asian malaria vector Anopheles dirus
Source: PLoS One. 2020 Apr 14;15(4):e0231251. doi: 10.1371/journal.pone.0231251 (PMC7156039; doi:10.1371/journal.pone.0231251)
Supplement: S1 Text — (DOCX) [file pone.0231251.s001.docx]

> *Anopheles dirus* *sensu stricto* ^[[1]](#footnote-1)^

GAATAAGTGTTGATATAAAATAGGATCTCCTCCACCAGCTGGATCAAAAAATGAAGTATTTAAATTTCGGTCTGTTAATAATATAGTAATTGCTCCTGCTAATACGGGTAAAGATAAAAGTAATAAAATTGCAGTAATTACAACTGATCAAACAAATAAAGGTATTCGATCTAAAGTAATTCCTGGTGATCGTATATTAATTACAGTAGTAATAAAATTTACTGCCCCTAAAATAGAAGAAATTCCTGCTAAATGTAAAGAAAAAATTGCTAAATCAACAGAAGCCCCGGCATGAGCAATTCCAGATGATAAAGGGGGATAAACTGTTCATCCTGTACCTGCTCCATTTTCTACTATACTTCTAGAAATTAAAAGTGTAAGTGAAGGAGGTAATATTCAAAAACTTATATTATTTATTCGAGGAAATGCTATATCTGGTGCTCCTAATATTAAAGGAACTAATCAATTTCCAAATCCTCCAATTATAATTGGTATAACTATAAAAAAAATTATGATAAATGCGTGAGCAGTAACAATAACATTATAAATTTGATCGTCTCCAATAAAAGCTCCTGGATGTCCTAATTCAGCTCGAATTAAGATTCTTAAAGAAGTTCCTACTATACCTGCTCAGGCTCCAAAAATAAAATATAAAGTA

1. All specimen (n= 5) gave the same sequence with no evidence of intra-specific variation at this locus. [↑](#footnote-ref-1)
