# Supplementary material for: Longevity of the insecticidal effect of three pyrethroid formulations applied to outdoor vegetation on a laboratory-adapted colony of the Southeast Asian malaria vector Anopheles dirus
Source: PLoS One. 2020 Apr 14;15(4):e0231251. doi: 10.1371/journal.pone.0231251 (PMC7156039; doi:10.1371/journal.pone.0231251)
Supplement: S1 Table — (DOCX) [file pone.0231251.s002.docx]

| **Number of specimen analyzed**^[[1]](#footnote-1)^ | **Identity** | **Accession** | **Genbank entry** |
| --- | --- | --- | --- |
| 5 | 99.80% | JX219732 | *Anopheles dirus sensu stricto* |

1. All specimen (n= 5) gave the same sequence with no evidence of intra-specific variation at this locus. [↑](#footnote-ref-1)
