# Supplementary material for: Longevity of the insecticidal effect of three pyrethroid formulations applied to outdoor vegetation on a laboratory-adapted colony of the Southeast Asian malaria vector Anopheles dirus
Source: PLoS One. 2020 Apr 14;15(4):e0231251. doi: 10.1371/journal.pone.0231251 (PMC7156039; doi:10.1371/journal.pone.0231251)
Supplement: Peer review history

# Peer review: Decision Letter 0

**DOI:** 10.1371/journal.pone.0231251.r001

**Authors:** Sean Michael Prager

20 Mar 2020

Longevity of the insecticidal effect of three pyrethroid formulations applied to outdoor vegetation on a laboratory-adapted colony of the Southeast Asian malaria vector Anopheles dirus

PONE-D-19-22372

Dear Dr. Chaumeau,

We are pleased to inform you that your manuscript has been judged scientifically suitable for publication and will be formally accepted for publication once it complies with all outstanding technical requirements.

Within one week, you will receive an e-mail containing information on the amendments required prior to publication. When all required modifications have been addressed, you will receive a formal acceptance letter and your manuscript will proceed to our production department and be scheduled for publication.

Shortly after the formal acceptance letter is sent, an invoice for payment will follow. To ensure an efficient production and billing process, please log into Editorial Manager at https://www.editorialmanager.com/pone/, click the "Update My Information" link at the top of the page, and update your user information. If you have any billing related questions, please contact our Author Billing department directly at authorbilling@plos.org.

If your institution or institutions have a press office, please notify them about your upcoming paper to enable them to help maximize its impact. If they will be preparing press materials for this manuscript, you must inform our press team as soon as possible and no later than 48 hours after receiving the formal acceptance. Your manuscript will remain under strict press embargo until 2 pm Eastern Time on the date of publication. For more information, please contact onepress@plos.org.

With kind regards,

Sean Michael Prager, Ph.D.

Academic Editor

PLOS ONE

Journal requirements:

Please ensure that the minor concerns raised by reviewer 2 are addressed in the final submission, including submitting sequence data to an appropriate public database.

Additional Editor Comments (optional):

Reviewers' comments:

Reviewer's Responses to Questions

**Comments to the Author**

1. Is the manuscript technically sound, and do the data support the conclusions?

The manuscript must describe a technically sound piece of scientific research with data that supports the conclusions. Experiments must have been conducted rigorously, with appropriate controls, replication, and sample sizes. The conclusions must be drawn appropriately based on the data presented. 

Reviewer #1: Yes

Reviewer #2: Yes

**********

2. Has the statistical analysis been performed appropriately and rigorously? 

Reviewer #1: Yes

Reviewer #2: Yes

**********

3. Have the authors made all data underlying the findings in their manuscript fully available?

The PLOS Data policy requires authors to make all data underlying the findings described in their manuscript fully available without restriction, with rare exception (please refer to the Data Availability Statement in the manuscript PDF file). The data should be provided as part of the manuscript or its supporting information, or deposited to a public repository. For example, in addition to summary statistics, the data points behind means, medians and variance measures should be available. If there are restrictions on publicly sharing data—e.g. participant privacy or use of data from a third party—those must be specified.

Reviewer #1: Yes

Reviewer #2: Yes

**********

4. Is the manuscript presented in an intelligible fashion and written in standard English?

PLOS ONE does not copyedit accepted manuscripts, so the language in submitted articles must be clear, correct, and unambiguous. Any typographical or grammatical errors should be corrected at revision, so please note any specific errors here.

Reviewer #1: Yes

Reviewer #2: Yes

**********

5. Review Comments to the Author

Please use the space provided to explain your answers to the questions above. You may also include additional comments for the author, including concerns about dual publication, research ethics, or publication ethics. (Please upload your review as an attachment if it exceeds 20,000 characters)

Reviewer #1: This study assesses the longevity of residual insecticides on vegetation under various natural climatic conditions.

The methods used are straightforward and well established. The modest conclusions are supported by the data presented. The choice of vegetation and mosquito highly appropriate for the setting. This type of highly focused work tailored for the specific conditions of the Thai Myanmar boarder is essential for the integrated elimination of malaria in this region. Well done nice work.

Reviewer #2: In the manuscript “Longevity of the insecticidal effect of three pyrethroid formulations applied to outdoor vegetation on a laboratory-adapted colony of the Southeast Asian malaria vector Anopheles dirus”, the authors investigated the persistence of insecticide residuals for three different insecticides, each with their own formulation. KD50 assays provide an interesting approach to assess insecticide resistance in the field, notably the selection of target site insensitivity in the VSSC, and was the assessment method used in their study. Since they used a lab strain (assumingly naive of insecticide application, or long lost to resistance having been lab reared for multiple decades without selection), an assessment of KD50 is properly the best test for which to assess the effect of strong insecticides, such as pyrethroids. The authors took care to establish a baseline response using technical grade impregnated papers so that a comparison could be made for the field-collected material and included these in each assay, which is critically important for accurate comparison.

Overall, the approach and results are sound. It is three different insecticides, each with their own formulation, which suggests that there should be some sort of comparison of which formulation, or which active ingredient is “best”, but the authors took care to not make these comparisons, which is the right approach for the conclusions. As the authors point out in their Table 1, there is seemingly a dearth of research to look at persistence of insecticide applications over time under field conditions, it’s certainly something that the NECE scientists have a strong priority for and this study definitely adds to the literature.

If the authors pursue future work to this, there are a few suggestions I would have. First would be to test formulations on multiple vegetation types or structures, so the results could similarly be inferred to other areas. Second, would be to do mock “spray cards”, to get an indication of droplet size and coverage patterns. This would be best used with cards and a fluorescent dye that could be visualized under a black light (similar to ones used to validate watermarks on money), so that between areas, the coverage could be held consistent.

A few fussy things:

The COI sequence needs to be submitted to NCBI and given an accession. Or submitted to boldsystems.org. Information for this can be found at: https://www.ncbi.nlm.nih.gov/guide/howto/submit-sequence-data/ or at http://www.boldsystems.org/

Table 1: spp. should be in regular (not italic) case.

Silicon oil- Dow 556?

nplr - needs citation - in R use: citation("nplr")

same for epitools ie: use citation("epitools")

For bamboo, put the species as well and (if possible a variety?). And if you can make a strong argument for “why bamboo”, it will really strengthen the manuscript. For example, if you have information on the acreage of bamboo surrounding peridomestic areas or knowledge of information such as bamboo is commonly associated with An. dirus populations, etc, it would be helpful for the reader.

For rearing please state the blood source and mention that the lab strain had never been selected for resistance if it had never been pressured.

**********

6. PLOS authors have the option to publish the peer review history of their article (what does this mean?). If published, this will include your full peer review and any attached files.

If you choose “no”, your identity will remain anonymous but your review may still be made public.

**Do you want your identity to be public for this peer review?** For information about this choice, including consent withdrawal, please see our Privacy Policy.

Reviewer #1: No

Reviewer #2: No

# Editor report: Acceptance letter

**DOI:** 10.1371/journal.pone.0231251.r002

**Authors:** Sean Michael Prager

30 Mar 2020

PONE-D-19-22372 

Longevity of the insecticidal effect of three pyrethroid formulations applied to outdoor vegetation on a laboratory-adapted colony of the Southeast Asian malaria vector *Anopheles dirus*

Dear Dr. Chaumeau:

I am pleased to inform you that your manuscript has been deemed suitable for publication in PLOS ONE. Congratulations! Your manuscript is now with our production department. 

If your institution or institutions have a press office, please notify them about your upcoming paper at this point, to enable them to help maximize its impact. If they will be preparing press materials for this manuscript, please inform our press team within the next 48 hours. Your manuscript will remain under strict press embargo until 2 pm Eastern Time on the date of publication. For more information please contact onepress@plos.org.

For any other questions or concerns, please email plosone@plos.org. 

Thank you for submitting your work to PLOS ONE.

With kind regards,

PLOS ONE Editorial Office Staff

on behalf of

Dr. Sean Michael Prager 

Academic Editor

PLOS ONE
